# UBE2S promotes the progression and Olaparib resistance of ovarian cancer through Wnt/β-catenin signaling pathway

**DOI:** 10.1186/s13048-021-00877-y

**Published:** 2021-09-17

**Authors:** Wenjing Hu, Min Li, Youguo Chen, Xinxian Gu

**Affiliations:** 1 Ultrasonic Diagnosis Room, Department of Obstetrics and Gynecology, The First Affiliated Hospital of Soochow University, 188 Shizi Street, Suzhou, 215006 China; 2 Ultrasound Department, Dushu Lake Hospital Affiliated to Soochow University, Suzhou, 215000 China; 3 Department of Obstetrics and Gynecology, The First Affiliated Hospital of Soochow University, 188 Shizi Street, Suzhou, 215006 China

**Keywords:** Ovarian Cancer, UBE2S, Olaparib, Wnt/β-catenin

## Abstract

**Background:**

Ovarian cancer is the most lethal gynecologic malignancy worldwide. Olaparib, an inhibitor of poly (ADP-ribose) polymerase (PARP), is becoming widely used in ovarian cancer treatment. The overall survival of ovarian cancer has not been significantly changed over the past decades and ovarian cancer has become increasingly resistant to the Olaparib. Ubiquitin-conjugating enzyme E2S (UBE2S) has been proved to promote malignant behaviors in many cancers. However, the function of UBE2S in the development and Olaparib resistance of ovarian cancer are unclear.

**Materials and methods:**

In this study, we detected the expression of UBE2S in normal fallopian tube (FT) and HGSOC tissues. A2780 and SKOV3 cells were stably transfected with PCMV-UBE2S, PCMV-UBE2S-C95S, UBE2S shRNAs, and negative controls. The CCK8 assay and clonogenic assay were conducted to analyze ovarian cancer proliferation and Olaparib resistance. The transwell assay was performed to determine the migration and invasion of ovarian cancer cells. The relative protein levels of the Wnt/β-catenin signaling pathway were tested using western blot. The ovarian cancer cells were treated with XAV-939 to investigate the role of Wnt/β-catenin signaling pathway in Olaparib resistance. Moreover, we repeated some above procedures in the xenograft model.

**Results:**

The results demonstrated that UBE2S was highly upregulated in HGSOC and that high UBE2S expression was correlated with poor outcomes in HGSOC. UBE2S promoted ovarian cancer proliferation and drived the migration and invasion of ovarian cancer cells. UBE2S activated the Wnt/β-catenin signaling pathway in ovarian cancer resulting in Olaparib resistance in vitro and in vivo. Furthermore, UBE2S enhanced the proliferation and Olaparib resistance of ovarian cancer in its enzymatic activity dependent manner.

**Conclusions:**

These data suggest a possible molecular mechanism of proliferation and metastasis of ovarian cancer and highlight the potential role of UBE2S as a therapeutic target in ovarian cancer.

**Supplementary Information:**

The online version contains supplementary material available at 10.1186/s13048-021-00877-y.

## Background

Ovarian cancer is the most common and fatal disease among a range of malignant tumors that affect the female reproductive tract. While advances in scientific discoveries have driven recent improvements in the prognosis of many other cancers, the death rate from ovarian cancer has stagnated since around 1980 [1, 2]. Obviously, ovarian cancer is not a unitary disease but rather encompasses a heterogenous group of malignancies. Epithelial malignancies are grouped as type I or type II based on clinicopathologic factors, of which type II epithelial carcinoma is the most common epithelial subtype with high grade, mainly including high-grade serous ovarian carcinoma (HGSOC) [3].

Ubiquitin conjugating enzyme E2S (UBE2S), also known as E2EPF, belongs to the E2 family of proteins [4–6]. Ubiquitination is a reversible physiological process in which ubiquitin is attached to substrate proteins. By regulating post-translational modifications of proteins, ubiquitination is involved in cell signaling, mitosis, endocytosis, and other cellular functions [4, 5]. UBE2S drives elongation of K11-linked ubiquitin chains by the Anaphase-Promoting Complex/Cyclosome (APC/C). APC/C is an E3 ubiquitin ligase that regulates mitosis and G1 [7]. UBE2S has been identified as an oncogene in EMC [5]. The UBE2S may be a novel marker contributing to lung cancer development, possibly through regulating canonical Wnt signaling [4]. UBE2S serves as an oncogene in hepatocellular carcinoma via enhancing the ubiquitination of p53 [6]. Currently, UBE2S upregulation is important in the prognosis of many human cancers. However, the biological function and prognostic significance of UBE2S in ovarian cancer are not fully understood.

Wnt/β-catenin signaling pathway is a highly evolutionarily conserved pathway that regulates key cellular functions including proliferation, differentiation, migration, genetic stability, apoptosis, and stem cell renewal [8]. Wnt signaling is critical to the activity of epithelial stem cells, hence changes in Wnt/β-catenin signaling are frequently observed in cancers [9]. In fact, the Wnt/β-catenin pathway has been shown to be involved in the carcinogenesis of many ovarian cancer subtypes including HGSOC [10–13].

In the present study, we analyzed the clinicopathological roles of UBE2S in HGSOC. We also investigated the molecules and pathways regulated by UBE2S. The results showed that UBE2S expression in HGSOC was up-regulated compared with that in normal tissues. High UBE2S expression is associated with poor prognosis in HGSOC patients. In addition, UBE2S promotes ovarian cancer cell proliferation, metastasis, and chemotherapeutic resistance.

## Methods

### Patients and tissue samples

A total of 120 cases of HGSOC tissues and 21 cases of FT tissues were collected at the First Affiliated Hospital of Soochow University between 2012 and 2016. The HGSOC tissues were acquired from primary ovarian cancer patients with no previous surgery or chemotherapy. The fallopian tube specimens were obtained from patients diagnosed with benign gynecologic tumors and received hysterectomy and bilateral salpingo-oophorectomy. All the experiments were approved by the Ethics Committee of the First Affiliated Hospital of Soochow University. The clinical pathologic characteristics of these patients are summarized in Table 1.

**Table 1:** Correlation between UBE2S expression and clinicopathological characteristics

Clinicopathological features	UBE2S expression		
	Low expression	High expression	*p* value
Ages (years)			
<56	17	24	0.974
≥ 56	33	46	
FIGO staging			
I + II	9	16	0.518
III + IV	41	54	
CA125 (U/ml)			
<600	21	24	0.373
≥ 600	22	36	
Lymph node metastasis			
Positive	12	26	0.017
Negative	25	18	
Omentum metastasis			
Positive	32	39	0.471
Negative	14	23	
Platinum Status			
Resistance	5	14	0.001
Sensitive	20	11	

### Cell lines and cell culture

A2780 cell line was purchased from Fuheng Biology (Shanghai, China). SKOV3 cell line was purchased from American Type Culture Collection (ATCC, Manassas, VA, USA). A2780 and SKOV3 cells were grown in RPMI 1640 and Dulbecco’s modified Eagle’s medium (DMEM), respectively, supplemented with 10% fetal bovine serum (FBS, Gibco, Grand Island, NY, USA). All cells were identified by STR profiling and cultured in a humidified incubator at 37 °C with 5% CO_2_.

### Antibodies and reagents

Antibodies for UBE2S (14115–1-AP) and β-actin (66009–1-Ig) were purchased from Proteintech (Wuhan, China). Antibodies for PARP (9532) and caspase-3 (9662) were obtained from Cell Signaling Technology (Danvers, MA, USA). Antibodies for c-Myc (ab32072), β-catenin (ab32572), and cyclin D1 (ab16663) were acquired from Abcam (Cambridge, UK). XAV-939 (S1180) and Olaparib (S1060) were obtained from Selleck Chemicals (Houston, TX, USA). Tosyl Arginine Methyl Ester (TAME, 172104) was purchased from Sigma-Aldrich (St. Louis, MO, USA).

### Plasmid construction and lentivirus production

The PCMV-UBE2S plasmid was produced by inserting the coding sequence of UBE2S genes into pLenti-C-Myc-DDK-IRES-Puro vector (PCMV, PS100069, OriGene, Rockville, MD, USA). pLKO.1 UBE2S shRNA1 (TRCN0000007672) and pLKO.1 UBE2S shRNA2 (TRCN0000007674) vectors were obtained from Sigma-Aldrich. Overlap extension PCR was used to generate a point mutant of UBE2S predicted active site cysteine mutated to serine (C95S) [7]. All the vectors were verified by DNA sequencing. Lentivirus particles were generated in HEK293T cells packaged by psPAX2 and pMD2.G. To acquire cell lines stably overexpression or knockdown of UBE2S, A2780, and SKOV3 cells were infected with lentivirus for 24 h and then selected for 10 days in a culture medium containing puromycin (P8833, Sigma-Aldrich).

### Western blot analysis

First, RIPA lysis buffer containing phenylmethyl sulfonyl fluoride (PMSF) was used to lyse tissue and cell samples mechanically. Protein concentration was determined by the bicinchoninic acid (BCA) method (Enhanced BCA Protein Assay Kit from Beyotime, Shanghai, China). Second, protein samples (20 or 40 μg/lane) were separated on a prepared 10% sodium dodecyl sulfate (SDS) -polyacrylamide gel and transferred to a polydisperse A-vinyl fluoride (PVDF) membrane (Millipore Corporation, Billerica, USA) electrophoretically. Then, the membrane was blocked with 5% non-fat milk for 1 h at room temperature, and the primary antibodies were incubated with the membrane overnight at 4 °C. Next, a horseradish peroxidase (HRP)-linked secondary antibody (Santa Cruz Biotechnology, Santa Cruz, CA, USA) was incubated with the membrane for 1 h at room temperature (RT) and washed with PBST. Finally, the membrane was examined with an enhanced chemiluminescence (ECL) kit (Thermo Fisher Scientific, Waltham, MA, USA). The protein band density was analyzed using ImageJ software (NIH, Bethesda, MA, USA).

### Quantitative real-time PCR (qRT-PCR)

The total RNA was collected from ovarian cancer cells using TRIzol reagent (15,596,018, Invitrogen). Total RNA was reverse transcribed to cDNA using PrimeScript RT reagent Kit (RR037A, TaKaRa, Kyoto, Japan). Real-time quantitative PCR (qPCR) was performed using SYBR Premix (RR420A, TakaRa) on the Applied Biosystems 7500 Fast system (Applied Biosystems, Waltham, MA, USA). The relative expression levels of specific genes were quantified using the ΔΔCt method and normalized to the β-actin gene. The sequences of the PCR primers were as follows: UBE2S, forward, 5′-CGATGGCATCAAGGTCTTTCCC-3′, and reverse, 5′-CAGCAGGAGTTTCATGCGGAAC-3′; β-actin, forward, 5′-CACCATTGGCAATGAGCGGTTC-3′, and reverse, 5′-AGGTCTTTGCGGATGTCCACGT-3′.

### Immunofluorescence staining

Cells with UBE2S overexpression and control cells were seeded on 1 cm glass slides. After being fixed with 4% paraformaldehyde (PFA) for 15 min at RT, the cells were permeabilized with Triton X-100 and blocked using normal goat serum. Then, the cells were stained with β-catenin antibody (1:100) overnight at 4 °C and corresponding secondary antibody (1:200) for 1 h at 37 °C. The nuclei were counterstained with DAPI. Images were observed and collected using the Olympus microscopy.

### Colony formation assay

About 600–800 treated cells were seeded in a 6-well plate and then cultured for up to 14 days. The colonies were fixed by paraformaldehyde for 15 min and then stained using crystal violet solution for 15 min. The number of colonies containing more than 50 cells was calculated and pictured.

### Tumor formation in vivo

The male BALB/c nude mice (4–6 weeks, 20–25 g) were obtained from NBRI of Nanjing University (Nanjing, China) and cultured in a pathogen-free facility. About 1 × 10^6^ cells with UBE2S overexpression or control cells were suspended with 100 μL of PBS and subcutaneously injected into the back of nude mice. The tumor volumes were calculated as the length×width^2^× 1/2. When the tumor reached about a volume of 100 mm^3^, the mice then received intraperitoneal injection of Olaparib or/and XAV-939 for 2 weeks. The mice were sacrificed and tumor nodules were harvested, measured, and photographed. All animal study procedures were approved by the Animal Care and Use Committee of Soochow University.

### Statistical analysis

All experiments were independently repeated at least three times. Statistical significance was assessed using the Student’s *t*-test for comparison between the two groups, and one-way ANOVA tests for three or more groups (GraphPad Software, La Jolla, CA, USA). Clinical characteristics were analyzed using Chi-squared test. Overall survival analysis was performed by Kaplan-Meier and log-rank tests. Images were processed using Adobe Photoshop CC 21.2.0 (Adobe, San Jose, CA, USA). *P* value < 0.05 was considered statistically significant and data were presented as the means ± SEM.

## Results

### UBE2S is highly expressed and correlates with poor outcome in HGSOC

**Fig. 1:**
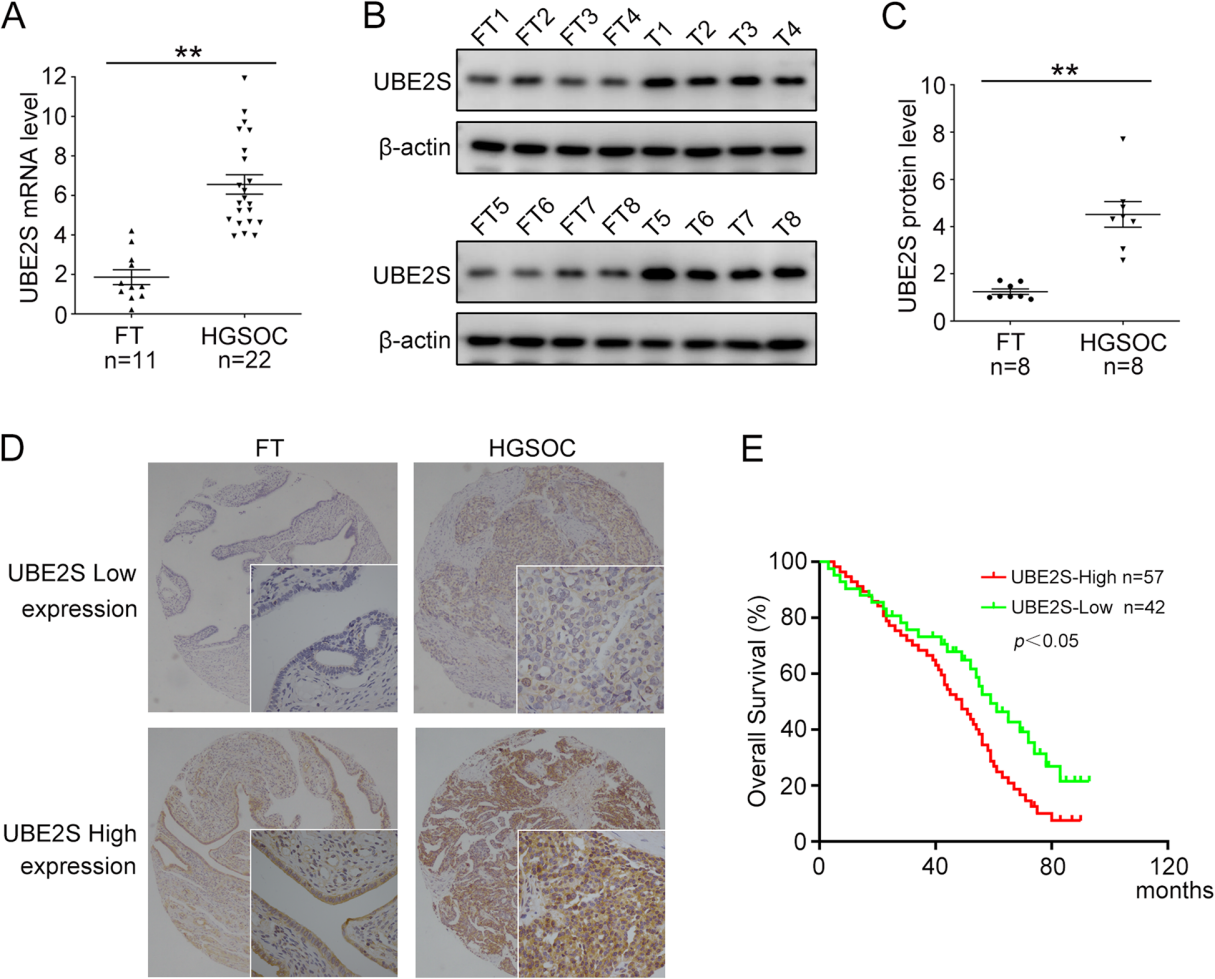
UBE2S is correlated with poor prognosis in HGSOC. **A** qRT-PCR was performed to detect the mRNA levels of UBE2S in normal fallopian tube (FT) and high-grade serous ovarian carcinoma (HGSOC) tissues. **B** Western blot analysis of the protein levels of UBE2S and β-actin in FT and HGSOC (T) tissues. **C** Quantification of relative protein levels of UBE2S in (**B**). **D** Representative images of IHC staining of UBE2S in HGSOC and FT tissues (Upper, 40×; Lower, 400×). **E** Overall survival (OS) analysis based on UBE2S expression in HGSOC patients. (Data are mean ± SEM, **p < 0.01)

To exam the expression of UBE2S, we explored the expression profile of UBE2S in the TCGA datasets and the UBE2S was highly expressed in various types of cancer (Additional file 1: Figure S1A). The results from TCGA datasets also showed that the expression of UBE2S was relatively high in ovarian cancer tissues compared with control tissues (Additional file 1: Figure S1B). Next, we identified the UBE2S expression level in the human HGSOC and normal FT tissues, and the results showed that the mRNA and protein levels of UBE2S in HGSOC were significantly elevated compared with that in FT tissues (Fig. 1A-C). All these results showed that the expression of UBE2S was extensively upregulated in HGSOC, indicating a possible role of UBE2S in ovarian cancer.

To assess the expression pattern and clinical significance of UBE2S in HGSOC, we examined the expression of UBE2S protein in HGSOC tissues and FT tissues using immunohistochemistry (IHC). The results were divided into 4 pictures based on tissue resource and UBE2S expression level (Fig. 1D). We also analyzed the correlation between UBE2S expression and clinicopathological characteristics (Table 1). The high expression of UBE2S in ovarian cancer was related with lymph node metastasis and platinum resistance, but not with age, FIGO staging, CA125 level, and peritoneal metastasis. Furthermore, Kaplan-Meier survival curves showed that the overall survival time of HGSOC patients with higher UBE2S expression was highly shorter than that of patients with lower UBE2S expression (Fig. 1E). We also identified the overall UBE2S expression in ovarian cancer patients using microarray data from Kaplan-Meier Plotter, which showed a shorter overall survival time (HR = 1.24, *p* = 0.0069) in patients with high UBE2S expression (Additional file 1: Figure S1C). These results suggest that high UBE2S expression correlates with poor outcome in HGSOC patients.

### UBE2S promotes ovarian cancer proliferation

**Fig. 2:**
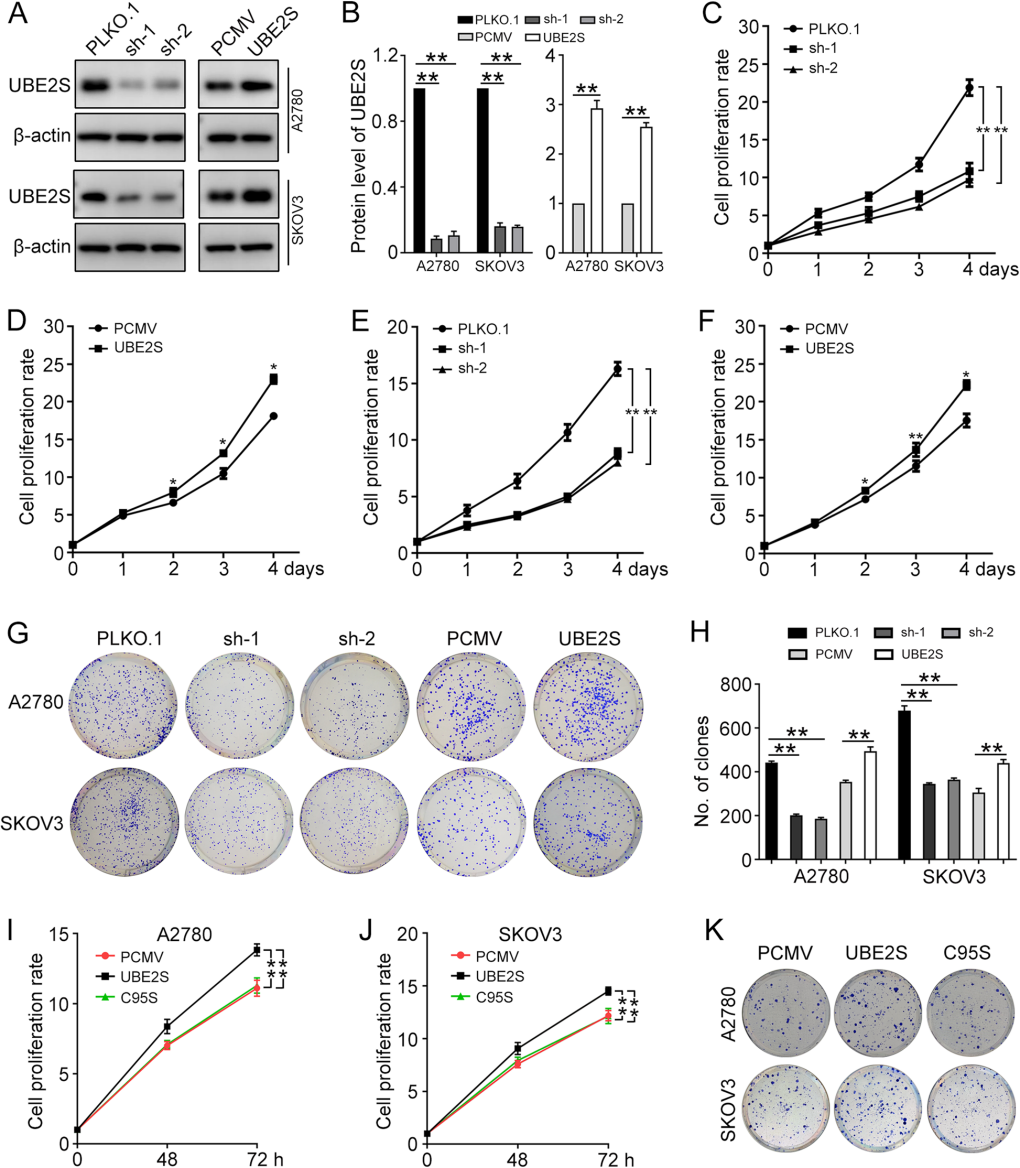
UBE2S promotes the proliferation of ovarian cancer cells. A2780 and SKOV3 cells were stably transfected with PLKO.1 (Ctr), UBE2S shRNA1 (sh-1), UBE2S shRNA2 (sh-2), PCMV (Ctr), and PCMV-UBE2S (UBE2S). **A** Western blot was performed to detect the protein level of UBE2S and β-actin in A2780 and SKOV3. **B** Quantification of relative protein levels of UBE2S in (**A**). The CCK8 assay was conducted to analyze the proliferation of A2780 (**C**, **D**) and SKOV3 (**E**, **F**) cells with UBE2S overexpression or knockdown. **G** Clonogenic assay was used to determine the colony formation efficiency of A2780 and SKOV3 cells. **H** Quantification of the number of clones in (**G**). A2780 and SKOV3 cells were stably transfected with PCMV, PCMV-UBE2S (UBE2S), and PCMV-UBE2S-C95S (C95S). **I** and **J** The CCK8 assay was conducted to analyze cell proliferation. **K** Clonogenic assay was used to determine the colony formation efficiency of A2780 and SKOV3 cells. Quantification of the number of clones was shown in Additional file 2. (Data are mean ± SEM, *p < 0.05, **p < 0.01, *n* = 3)

To exam the biological significance of UBE2S in ovarian cancer, we stably transfected A2780 and SKOV3 cells with PLKO.1 (Ctr), UBE2S shRNA1 (sh-1), UBE2S shRNA2 (sh-2), PCMV (Ctr), and PCMV-UBE2S (UBE2S). The western blot was performed to detect the protein levels of UBE2S and β-actin in cells, which assess the expression of UBE2S after the transfection (Fig. 2A and B). Then, the Cell Counting Kit-8 (CCK8) assay and clonogenic assay were performed and showed that higher expression of UBE2S promotes the proliferation and colony formation efficiency of A2780 and SKOV3 cells (Fig. 2C-H). These data revealed that the UBE2S plays a significant role in ovarian cancer proliferation.

To further investigate whether the function of UBE2S depended on its catalytic activity, a point mutant of the predicted active site cysteine mutated to serine (C95S) was generated [7]. The CCK8 assay and clonogenic assay showed that C95S mutant of UBE2S failed to improve the proliferative ability of A2780 and SKOV3 cells (Fig. 2I-K). Thus, the enzymatic activity of UBE2S is required to promote ovarian cancer proliferation.

### UBE2S promotes the migration and invasion of ovarian cancer cells

**Fig. 3:**
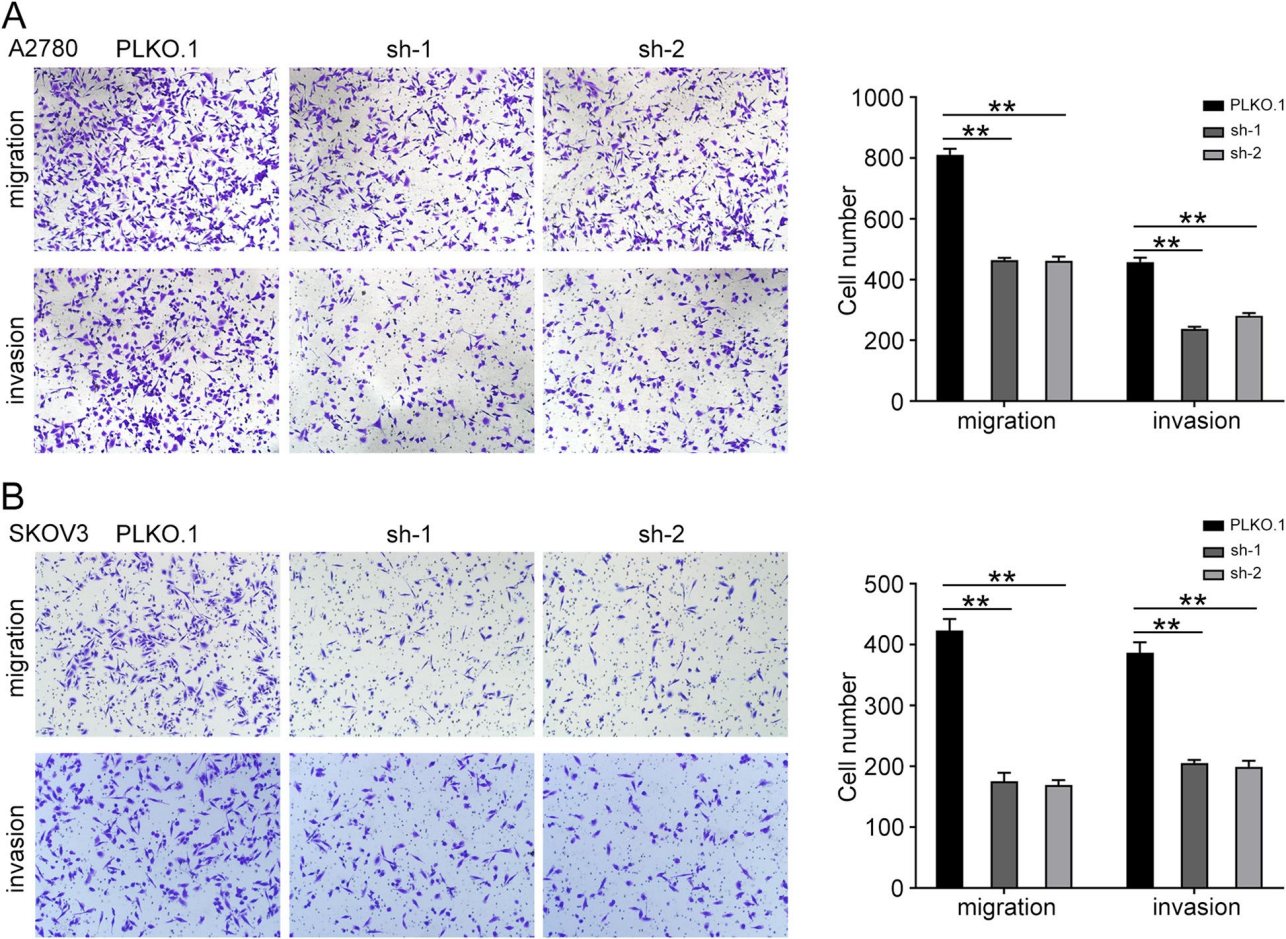
UBE2S promotes the metastasis of ovarian cancer cells. **A** The transwell assay was performed to determine the migration and invasion of A2780 cells with UBE2S knockdown. **B** The transwell assay was performed to determine the migration and invasion of SKOV3 cells with UBE2S knockdown. (Data are mean ± SEM, *p < 0.05, **p < 0.01, *n* = 3)

The transwell assay was used to analyze the effects of UBE2S on the migration and invasion of ovarian cancer cells. The results showed that overexpression of UBE2S significantly improves the migration and invasion power, while the inhibition of UBE2S significantly impaired the migration and invasion ability of A2780 and SKOV3 cells (Fig. 3A and B). These findings suggested that UBE2S promoted migration and invasion of ovarian cancer cells.

### UBE2S confers to Olaparib resistance in ovarian cancer cells

**Fig. 4:**
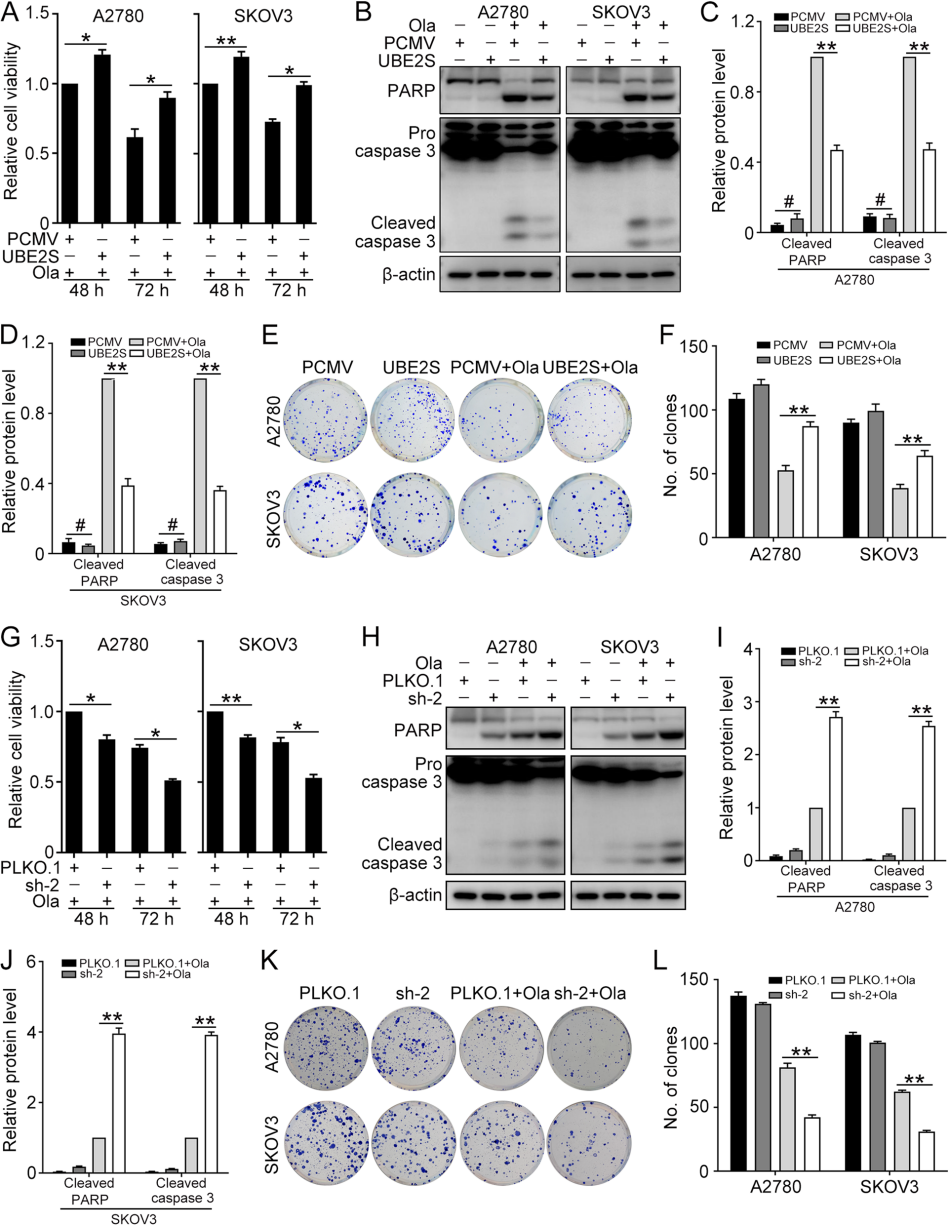
UBE2S enhances Olaparib resistance in ovarian cancer cells. A2780 and SKOV3 cells transfected with PCMV and PCMV-UBE2S (UBE2S) were treated with Olaparib (A2780, 15 μM; SKOV3, 30 μM) for 48 h and 72 h. **A** The CCK8 assay was performed to detect cell viability. **B** The protein level of PARP, cleaved caspase 3, and β-actin were detected by western blot. **C** and **D** Quantification of relative protein levels in (**B**). **E** Clonogenic assay was performed to determine the colony formation efficiency of A2780 and SKOV3 cells. **F** Quantification of the number of clones in (**E**). A2780 and SKOV3 cells transfected with PLKO.1 and UBE2S shRNA2 (sh-2) were treated with Olaparib (A2780, 15 μM; SKOV3, 30 μM) for 48 h and 72 h. **G** The CCK8 assay was performed to detect cell viability. **H** The protein level of PARP, cleaved caspase 3, and β-actin were detected by western blot. **I** and **J** Quantification of relative protein levels in (**H**). **K** Clonogenic assay was performed to determine the colony formation efficiency of A2780 and SKOV3 cells. **L** Quantification of the number of clones in (**K**). (Data are mean ± SEM, ^#^*p* > 0.05, *p < 0.05, **p < 0.01, *n* = 3)

To investigate the function of UBE2S on Olaparib resistance of ovarian cancer cells. A2780 and SKOV3 cells transfected with PCMV and PCMV-UBE2S were treated with Olaparib (A2780, 15 μM; SKOV3, 30 μM). The CCK8 assay showed that the cell viability was higher in cells with PCMV-UBE2S compared with that in cells with PCMV (Fig. 4A). The western blot was performed to determine the protein levels of PARP, cleaved caspase 3, and β-actin in the experiment and control group (Fig. 4B). The quantification results showed that the overexpression of UBE2S significantly decreased the cell apoptosis and thus increased the Olaparib resistance in ovarian cancer (Fig. 4C and D). Likewise, the clonogenic assay showed that overexpression of UBE2S significantly decreased the Olaparib sensitivity in ovarian cancer cells, in which the cell growth was obviously enhanced (Fig. 4E and F). As shown in Fig. 4G, the CCK8 assay showed that UBE2S knockdown remarkably increased the sensitivity of A2780 and SKOV3 cells to Olaparib. Besides, knockdown of UBE2S increased the level of cleaved caspase-3 and cleaved PARP induced by Olaparib (Fig. 4H-J). The clonogenic assay verified that UBE2S knockdown decreased colony-forming capacity of cells upon Olaparib treatment (Fig. 4K and L). These findings confirmed that UBE2S confers to the Olaparib resistance in ovarian cancer cells.

**Fig. 5:**
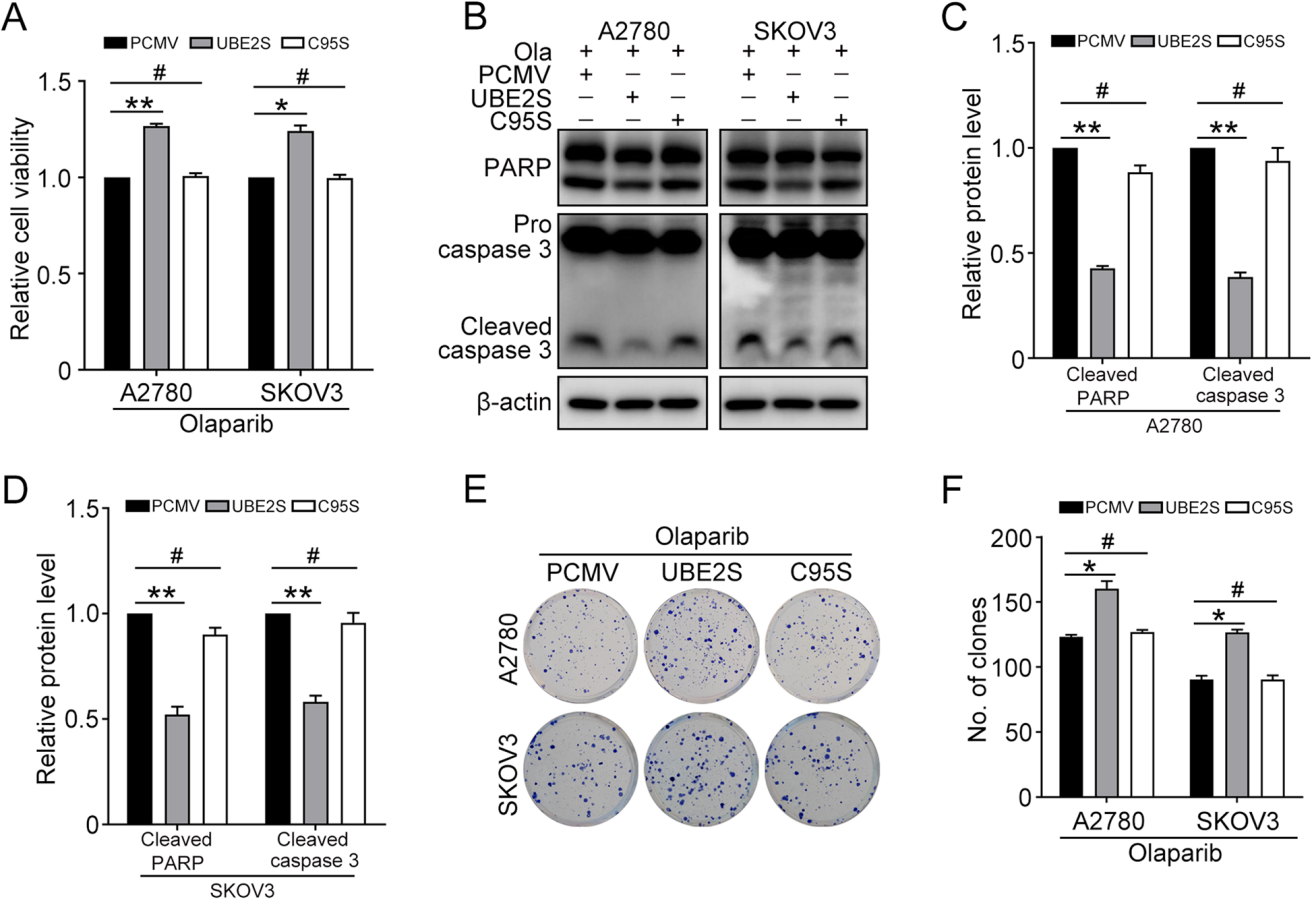
UBE2S promotes Olaparib resistance via its enzymatic activity. A2780 and SKOV3 cells transfected with PCMV, PCMV-UBE2S (UBE2S), and PCMV-UBE2S-C95S (C95S) were treated with Olaparib (A2780, 15 μM; SKOV3, 30 μM) for 48 h. **A** The CCK8 assay was performed to detect cell viability. **B** The protein levels of PARP, cleaved caspase 3, and β-actin were detected by western blot. **C** and **D** Quantification of relative protein levels in (**B**). **E** Clonogenic assay was performed to determine the colony formation efficiency of A2780 and SKOV3 cells. **F** Quantification of the number of clones in (**E**). (Data are mean ± SEM, *p < 0.05, **p < 0.01, *n* = 3)

Next, to explore the function of UBE2S C95S mutant in the Olaparib resistance, A2780 and SKOV3 cells transfected with PCMV, PCMV-UBE2S (UBE2S), PCMV-UBE2S C95S (C95S) were challenged with Olaparib (A2780, 15 μM; SKOV3, 30 μM) for 48 h. Different from UBE2S group, the CCK8 results suggested that cells transfected with C95S could not induce Olaparib resistance (Fig. 5A). Besides, C95S had no effect on the cell apoptosis induced by Olaparib in A2780 and SKOV3 cells (Fig. 5B-D). The clonogenic assay reached a similar conclusion (Fig. 5E and F). Therefore, the Olaparib resistance function of UBE2S relied on its enzymatic activity.

### UBE2S activates Wnt/β-catenin signaling pathway in ovarian cancer

**Fig. 6:**
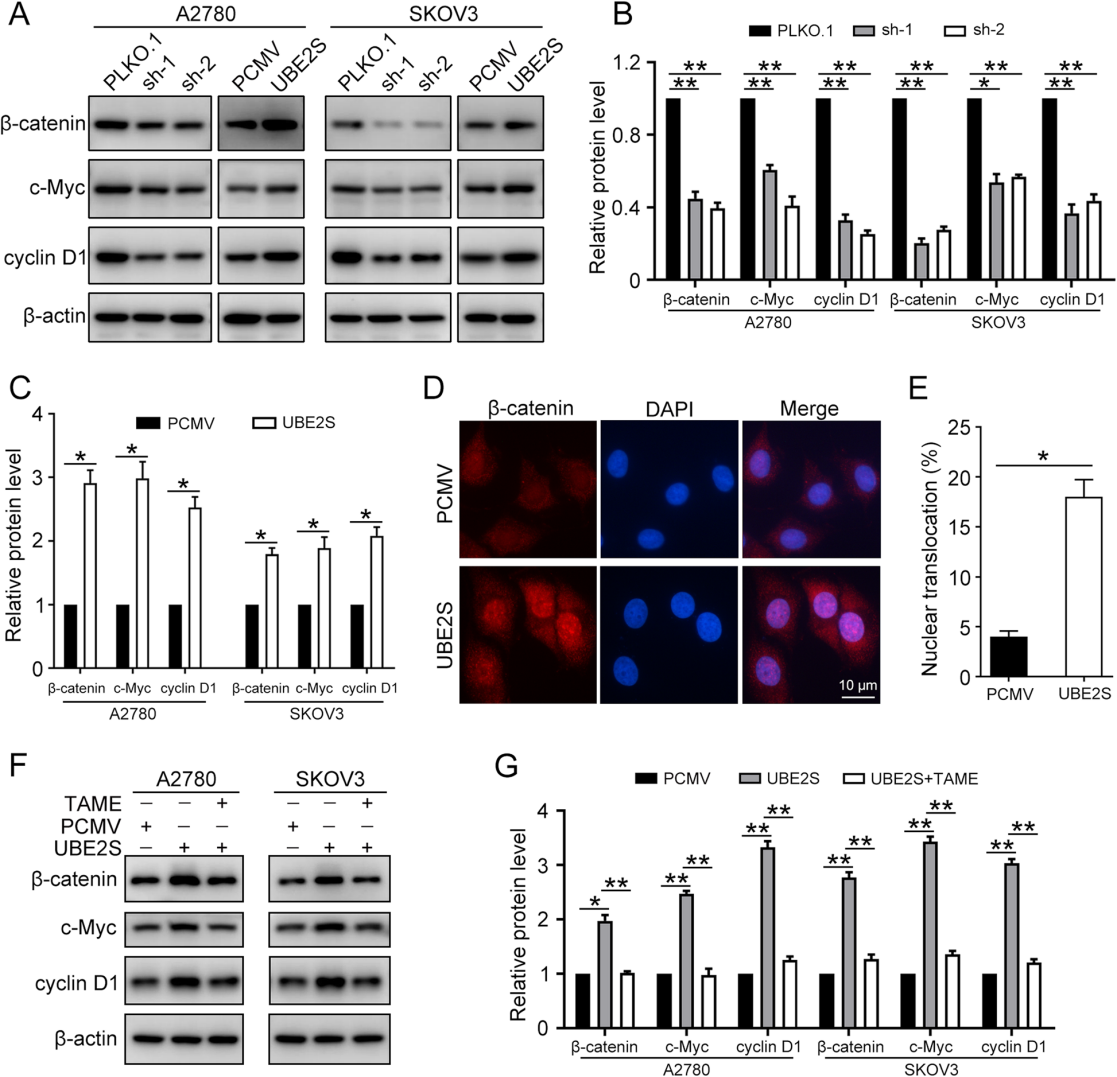
UBE2S induces Wnt/β-catenin activation in ovarian cancer cells. **A** Western blot was conducted to detect the protein levels of β-catenin, c-Myc, cyclin D1, and β-actin in A2780 and SKOV3 cells transfected with PLKO.1 (Ctr), UBE2S shRNA1 (sh-1), UBE2S shRNA2 (sh-2), PCMV (Ctr), and PCMV-UBE2S (UBE2S). **B** and **C** Quantification of relative protein levels of β-catenin, c-Myc, and cyclin D1 in (**A**). **D** Immunofluorescence staining was performed to detect the nuclear translocation of β-catenin (Red) in A2780 cells with UBE2S overexpression. Nuclei were counterstained with DAPI (Blue). Scale bar: 10 μm. **E** Quantification of the percentage of β-catenin nuclear translocation in (**D**). **F** A2780 and SKOV3 cells transfected with PCMV and PCMV-UBE2S (UBE2S) were treated with or without TAME (10 μM) for 24 h. Western blot was conducted to detect the protein levels of β-catenin, c-Myc, cyclin D1, and β-actin. **G** Quantification of the relative protein levels in (**F**). (Data are mean ± SEM, *p < 0.05, **p < 0.01, *n* = 3)

To investigate the relationship between UBE2S and Wnt/β-catenin signaling pathway, the A2780 and SKOV3 cells were transfected with PLKO.1 (Ctr), UBE2S shRNA1 (sh-1), UBE2S shRNA2 (sh-2), PCMV (Ctr), and PCMV-UBE2S (UBE2S). Then, the western blot was performed to detect the protein levels of β-catenin, c-Myc, cyclin D1, and β-actin (Fig. 6A-C). Our results showed that the overexpression of UBE2S significantly increased the relative protein level of Wnt/β-catenin signaling pathway, while the UBE2S knockdown decreased that. Furthermore, the immunofluorescence staining demonstrated that upregulation of UBE2S promotes the β-catenin accumulation in cytoplasm and transportation into nucleus (Fig. 6D and E). When the concentration of β-catenin in the cytoplasm reaches a certain level, it can transfer into the nucleus. This is a sign of the activation of the Wnt/β-catenin signaling pathway. Thus, we speculated that UBE2S activates the Wnt/β-catenin signaling pathway in ovarian cancer.

Previous report demonstrated that UBE2S interacted with APC/C complex to ubiquitinate the K19 residue of β-Catenin to prevent its degradation [14]. To clarify whether UBE2S activates Wnt/β-catenin signaling pathway through APC/C, an APC/C specific inhibitor, TAME, was used to block the function of APC/C in ovarian cancer cells. Western blots results showed that UBE2S overexpression failed to increase the protein level of Wnt/β-catenin signaling pathway in the presence of TAME (Fig. 6F and G). Thus, UBE2S promoted the activation of Wnt/β-catenin signaling pathway through APC/C.

### UBE2S promotes Olaparib resistance through Wnt/β-catenin signaling pathway in ovarian cancer

**Fig. 7:**
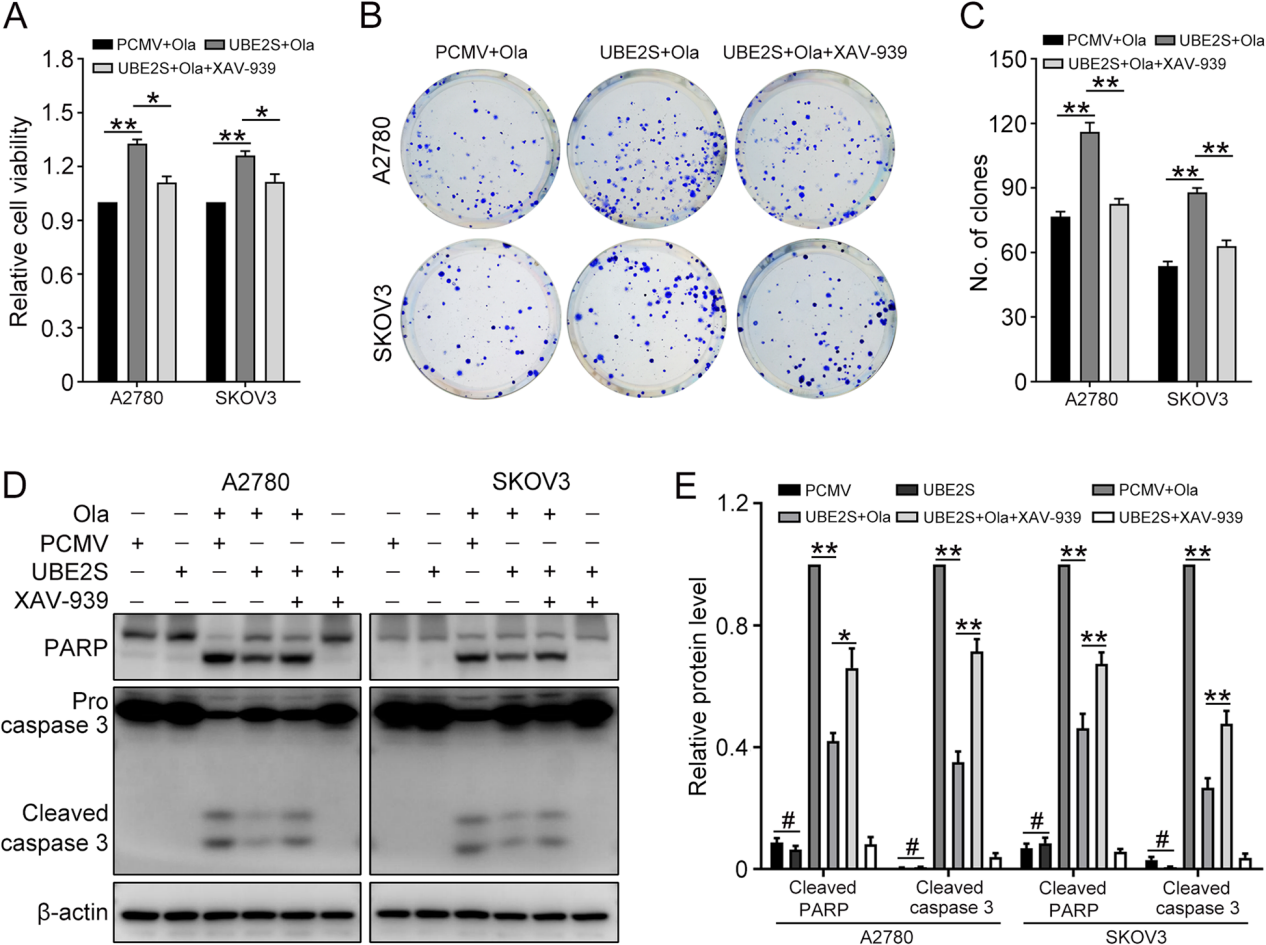
UBE2S confers to Olaparib resistance through Wnt/β-catenin in ovarian cancer cells. **A** The CCK8 assay was performed to determine the cell viability in ovarian cancer cells treated with Olaparib (A2780, 15 μM; SKOV3, 30 μM) or XAV-939 (10 μM) for 48 h. **B** Clonogenic assay was performed to determine the colony formation efficiency of A2780 and SKOV3 cells treated with Olaparib and XAV-939. **C** Quantification of the number of clones in (**B**). **D** The protein of PARP, cleaved caspase 3, and β-actin were detected by western blot in ovarian cancer cells treated with Olaparib (A2780, 15 μM; SKOV3, 30 μM) or XAV-939 (10 μM) for 48 h. **E** Quantification of the protein levels of cleaved PARP and cleaved caspase 3 in (**D**). (Data are mean ± SEM, ^#^*p* > 0.05, *p < 0.05, **p < 0.01, *n* = 3)

Considering that UBE2S was involved in the activation of Wnt/β-catenin signaling pathway and the Olaparib resistance in ovarian cancer, we examined the underlying relationship between them. XAV-939 was used to selectively inhibit the Wnt/β-catenin signaling pathway. The CCK8 assay showed that the relative cell viability was increased with Wnt/β-catenin signaling pathway interdiction (Fig. 7A). The clonogenic assay demonstrated the same results (Fig. 7B and C). Then the apoptosis-associated protein expression level was analyzed through western blot (Fig. 7D and E). The results showed that Olaparib promoted the expression of pro-apoptotic proteins Cleaved PARP and Cleaved Caspase 3, while UBE2S overexpression could inhibit the promotion effects. What’s more, the interruption of the Wnt/β-catenin signaling pathway attenuated the inhibition function of UBE2S. All these findings confirmed that UBE2S confers ovarian cancer cells to Olaparib resistance through Wnt/β-catenin signaling pathway.

### UBE2S promotes Olaparib resistance in vivo

**Fig. 8:**
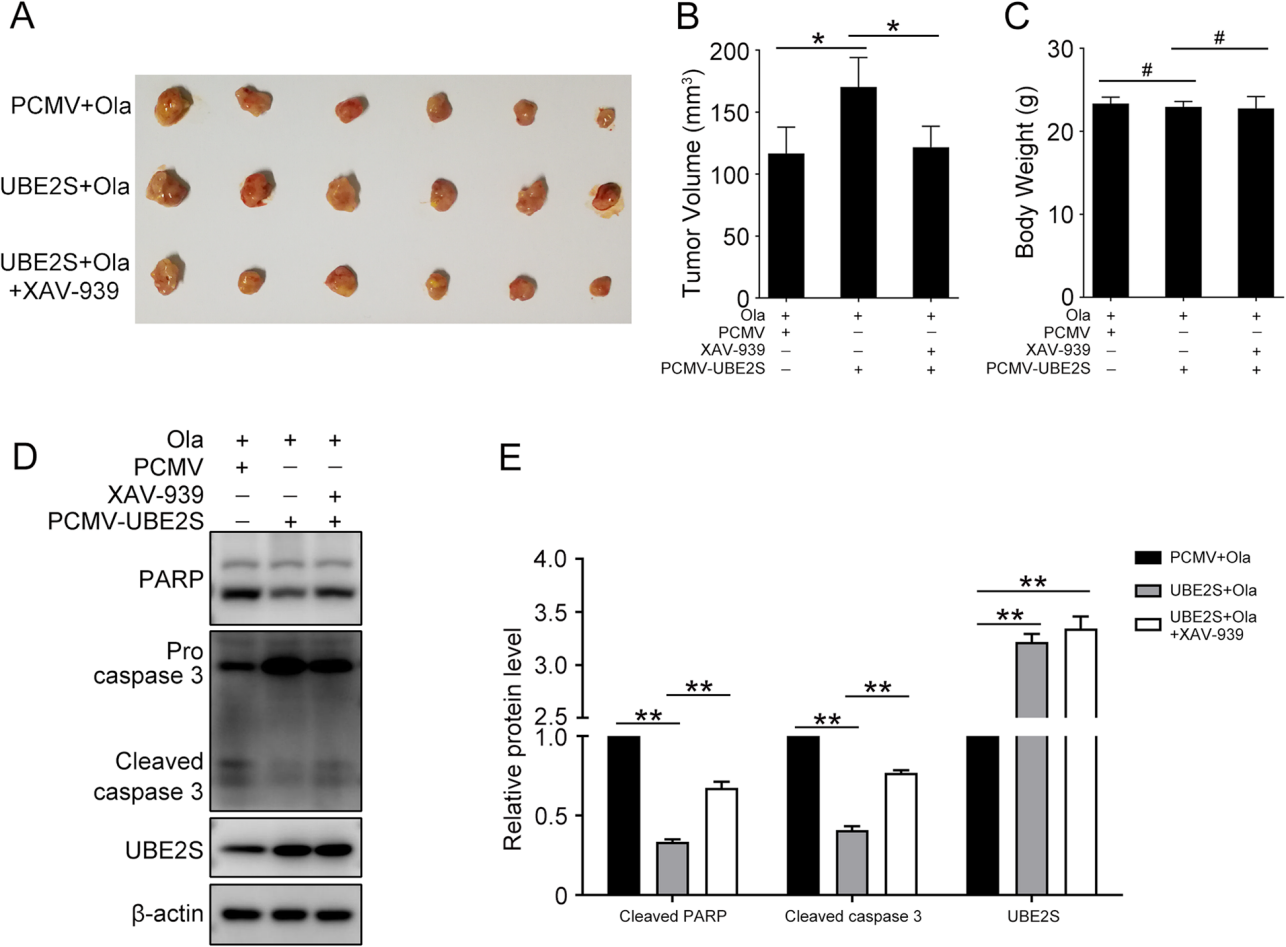
UBE2S induces Olaparib resistance through Wnt/β-catenin in xenograft model. 6 weeks old mice were subcutaneously injected with A2780 cells. When the tumor volumes reached about 50–100 mm^3^, tumor-bearing mice then received intraperitoneal injection of Olaparib (20 mg/kg, once a day) or/and XAV-939 (15 mg/kg, once a day). Two weeks after injection, the mice were sacrificed to harbor tumor tissues which were photographed. **A** Tumors from each group were shown. **B** The tumor volumes of each group. **C** The body weight of each group. **D** Western blot was performed to detect the protein levels of PARP, cleaved caspase 3, UBE2S, and β-actin in tumor tissues. **E** Quantification of the protein levels in (**D**). (Data are mean ± SEM, ^#^*p* > 0.05, *p < 0.05, **p < 0.01, *n* = 6)

Although in vitro studies have confirmed the unique role of UBE2S in HGSOC, we also explore the importance of UBE2S to promote Olaparib resistance in a xenograft mouse model. The tumor-bearing mouse was received intraperitoneal injection of Olaparib or/and XAV-939 for 2 weeks (Fig. 8A-C). The tumor tissue volumes of each group showed that, as same as in vitro, UBE2S induced Olaparib resistance through Wnt/β-catenin signaling pathway in vivo. Likewise, the western blot results showed that UBE2S downregulated the level of pro-apoptotic proteins Cleaved Caspase 3 and Cleaved PARP, while XAV-939 upregulated that (Fig. 8D and E). Collectively, these findings revealed that UBE2S promotes Olaparib resistance in vivo.

## Discussion

In previous studies, UBE2S has been demonstrated to exert oncogenic activities in several human malignancies [5, 6, 15–18]. This study revealed for the first time that overexpression, biological significance, and functional mode of UBE2S in ovarian cancer. In the present study, compared with FT tissues, HGSOC tissues showed a significant overexpression of UBE2S. Also, higher UBE2S expression was related with positive lymph node metastasis, platinum resistance, and shorter overall survival time. Our results indicated that UBE2S had the potential to be a novel biomarker for the development and drug resistance in ovarian cancer.

Recent study reported that UBE2S expression positively correlated with malignancy and resistance to chemo-radiotherapy in glioma [19]. UBE2S was demonstrated to promote the epithelial-mesenchymal transition (EMT) through the VHL/HIF-1α/STAT3 signaling pathway in pancreatic ductal adenocarcinoma [20]. The ectopic expression of UBE2S promoted cell proliferation and migration via regulating SOX6/β-Catenin in endometrial cancer (EMC) [5]. In the present study, we showed that overexpression of UBE2S promotes the proliferation and metastasis of ovarian cancer cells, while knockdown of UBE2S resulted in totally opposite phenotypes. These results revealed that UBE2S is an oncogene in ovarian cancer. Besides, C95S mutant of UBE2S failed to confer the ovarian cancer cells to proliferation, indicating that the function of UBE2S depended on its catalytic activity.

Poly (ADP-ribose) polymerase inhibitors (PARPis) are therapies approved for the treatment of epithelial ovarian cancer, and Olaparib was the first PARP inhibitor introduced in clinical practice [21]. The most common avenue of PARP inhibitor resistance is the restoration of the HR pathway [22]. Wnt/β-catenin signaling is known to play an important role in various aspects of physiology and pathology [23]. Further, dysregulated Wnt/β-catenin signaling has been shown to cause drug resistance in pancreatic cancer. Blocking Wnt/β-catenin signaling decreases taxane-resistant cells and can increase the sensitivity of taxane treatment in pancreatic cancer patients [24]. UBE2S mediates tumor progression through regulating Wnt/β-catenin signaling in colorectal cancer [14] and non-small cell lung cancer [4]. Here, our study showed that UBE2S upregulation confers to Olaparib resistance through activating the Wnt/β-catenin signaling pathway in vitro and in vivo. Besides, inhibition of Wnt/β-catenin using a specific inhibitor reversed the Olaparib resistance function of UBE2S overexpression. Thus, targeted inhibition of UBE2S might overcome Olaparib resistance in ovarian cancer.

The Anaphase-Promoting Complex/Cyclosome (APC/C) is an E3 ubiquitin ligase that regulates mitosis and G1, and UBE2S is an important component of the APC/C ubiquitination pathway [7, 25]. UBE2S was proved to promoted β-Catenin accumulation through K11-linked polyubiquitination in an APC/C-dependent manner. In the current study, we demonstrated that TAME, an APC/C specific inhibitor, restrained the activation of Wnt/β-catenin signaling induced by UBE2S overexpression. Therefore, these results showed that UBE2S activated Wnt/β-catenin signaling through interacting with APC/C in ovarian cancer.

## Conclusion

In the current study, we found the overexpression of UBE2S in HGSOC and its correlation with poor outcome. UBE2S plays a significant role in proliferation, migration, and invasion of HGSOC. UBE2S activates the Wnt/β-catenin signaling pathway in ovarian cancer to inhibit apoptosis, conferring Olaparib resistance in ovarian cancer cells. Finding a therapeutic strategy tailored to special biomarker would be a crucial advance in the systemic treatment of ovarian cancer. Our study indicates that UBE2S may be a therapeutic target in ovarian cancer, suggesting future directions to enhance the survival of ovarian cancer.

## Supplementary Information

**Additional file 1: Figure S1.** The expression of UBE2S is extensively higher in ovarian cancer. (A) TCGA datasets (http://ualcan.path.uab.edu/) showed that UBE2S was highly expressed in various types of cancer. (B) TCGA datasets (http://gepia.cancer-pku.cn/) showed that the expression of UBE2S was relatively high in ovarian cancer (OC) tissues compared with fallopian tube (FT) tissues. (C) Overall survival (OS) of UBE2S in ovarian cancer patients using microarray data from Kaplan-Meier Plotter (http://www.kmplot.com/analysis).

**Additional file 2: Figure S2.** Quantification of the number of clones in Fig. 2K. (Data are mean ± SEM, *p < 0.05, **p < 0.01, *n* = 3).

## Data Availability

The datasets used and/or analyzed during the current study are available from the corresponding author on reasonable request.

## Abbreviations

ATCC: American Type Culture Collection
APC/C: Anaphase-Promoting Complex/Cyclosome
CCK8: Cell Counting Kit-8
FBS: Fetal bovine serum
FT: Fallopian tube
HGSOC: High-grade serous ovarian carcinoma
IHC: Immunohistochemistry
PARP: Poly (ADP-ribose) polymerase
PARPi: Poly (ADP-ribose) polymerase inhibitor
PCMV: pLenti-C-Myc-DDK-IRES-Puro vector
TAME: Tosyl Arginine Methyl Ester
UBE2S: Ubiquitin-conjugating enzyme E2S
