# Supplementary material for: UBE2S promotes the progression and Olaparib resistance of ovarian cancer through Wnt/β-catenin signaling pathway
Source: J Ovarian Res. 2021 Sep 17;14:121. doi: 10.1186/s13048-021-00877-y (PMC8447717; doi:10.1186/s13048-021-00877-y)
Supplement: Supplementary file 1 — Additional file 1: Figure S1. The expression of UBE2S is extensively higher in ovarian cancer. (A) TCGA datasets (http://ualcan.path.uab.edu/) showed that UBE2S was highly expressed in various types of cancer. (B) TCGA datasets (http://gepia.cancer-pku.cn/) showed that the expression of UBE2S was relatively high in ovarian cancer (OC) tissues compared with fallopian tube (FT) tissues. (C) Overall survival (OS) of UBE2S in ovarian cancer patients using microarray data from Kaplan-Meier Plotter (http://www.kmplot.com/analysis). [file 13048_2021_877_MOESM1_ESM.docx]

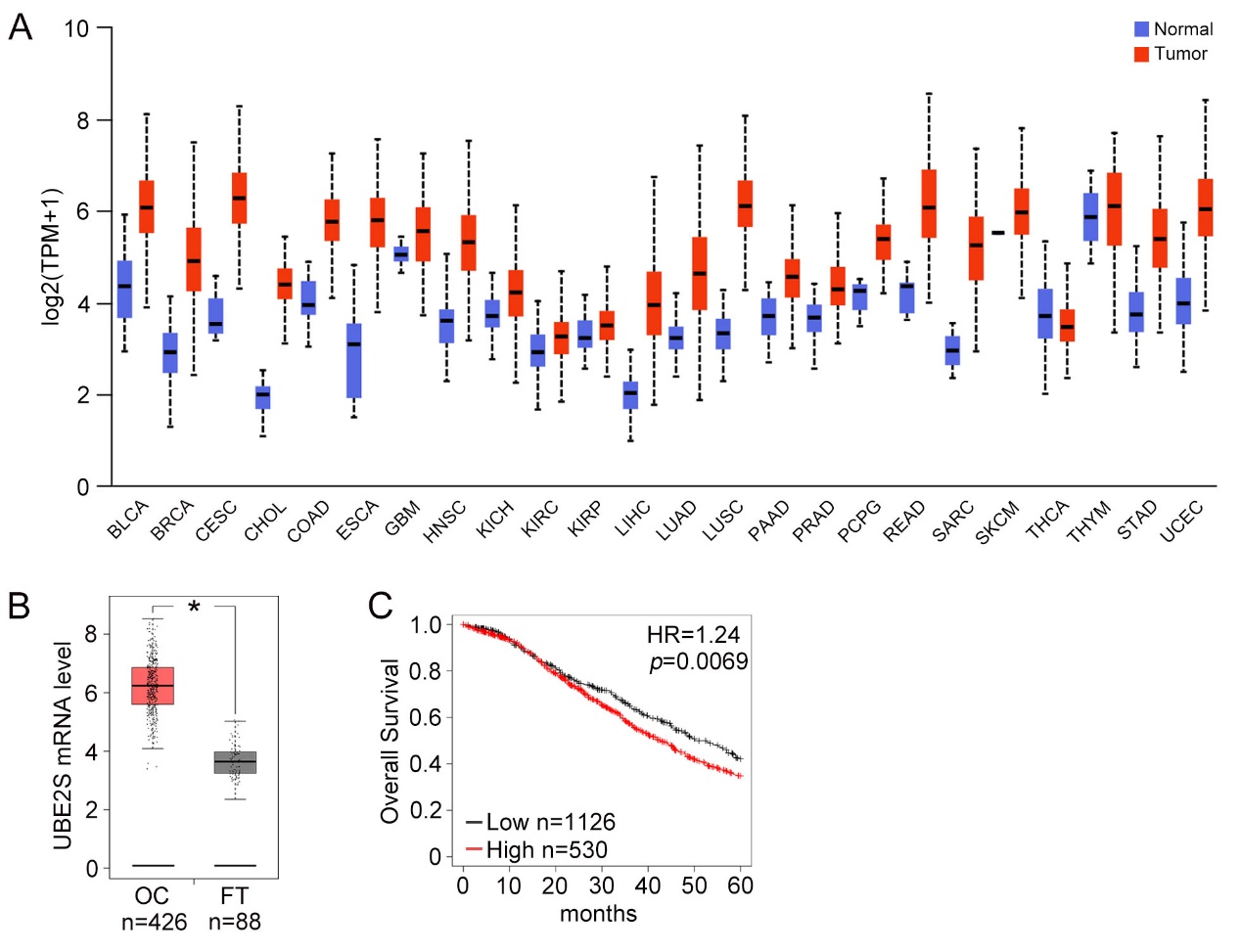


Figure S1. The expression of UBE2S is extensively higher in ovarian cancer. (A) TCGA datasets (http://ualcan.path.uab.edu/) showed that UBE2S was highly expressed in various types of cancer. (B) TCGA datasets (http://gepia.cancer-pku.cn/) showed that the expression of UBE2S was relatively high in ovarian cancer (OC) tissues compared with fallopian tube (FT) tissues. (C) Overall survival (OS) of UBE2S in ovarian cancer patients using microarray data from Kaplan-Meier Plotter (http://www.kmplot.com/analysis).
