# Supplementary figures and images for: UBE2S promotes the progression and Olaparib resistance of ovarian cancer through Wnt/β-catenin signaling pathway
Source: J Ovarian Res. 2021 Sep 17;14:121. doi: 10.1186/s13048-021-00877-y (PMC8447717; doi:10.1186/s13048-021-00877-y)

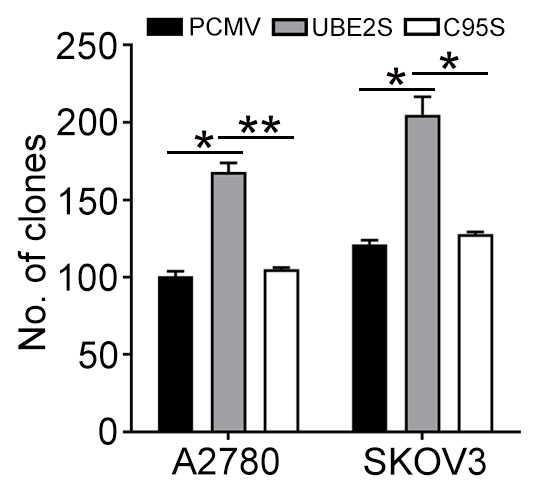


Figure S2. Quantification of the number of clones in Figure 2K. (Data are mean ± SEM, **p* < 0.05, ***p* < 0.01, n = 3).

Supplement: Supplementary file 2 — Additional file 2: Figure S2. Quantification of the number of clones in Fig. 2K. (Data are mean ± SEM, *p < 0.05, **p < 0.01, n = 3). [file 13048_2021_877_MOESM2_ESM.docx]
